# Profiles of Social Capital and the Association With Depressive Symptoms Among Multicultural Adolescents in Korea: A Latent Profile Analysis

**DOI:** 10.3389/fpubh.2022.794729

**Published:** 2022-03-08

**Authors:** Eunjoo Kim, Min Kyung Song

**Affiliations:** ^1^Research Institute of Nursing Science, College of Nursing, Seoul National University, Seoul, South Korea; ^2^Department of Nursing, College of Medicine, University of Ulsan, Ulsan, South Korea

**Keywords:** multicultural adolescents, social capital, latent profile analysis (LPA), Korea (Rep.), depressive symptoms

## Abstract

Low social capital has been reported to be associated with depression in adolescents. In general, adolescents with immigrant backgrounds lack social capital. By employing a latent profile analysis (LPA) for the specification of social capital among multicultural adolescents, depression interventions can be explored through the framework of social capital. The purpose of this study was to identify distinct latent profiles of social capital and explore the depressive symptoms of multicultural adolescents in those profiles. Data of 1,244 adolescents from the Multicultural Adolescents Panel Survey (MAPS) were used, which was conducted for 3^rd^-year middle school students in 2016. An LPA was used to identify profiles by different social capital classes and Quade's non-parametric ANCOVA was used to confirm the differences in depressive symptoms between profiles. Latent profile analysis indicated four classes. Analysis revealed that there were differences in the level of depressive symptoms according to the social capital sub-profiles (*F* = 44.42, *p* < 0.001). Class 1 had the lowest level of depressive symptoms (13.91 ± 4.43) and Class 4 had the highest level (18.07 ± 5.25). The depressive symptoms scores of Classes 2 and 3 were 16.49 ± 5.27 and 16.74 ± 4.95, respectively. These findings provide insight into the interplay between social capital and depressive symptoms among multicultural adolescents. Support in consideration of differences in social capital is needed to reduce depression among multicultural adolescents.

## Introduction

International migration is accelerating worldwide (1). The World Migration Report 2020 estimates the global number of international migrants to be approximately 272 million, exceeding the 2050 projection of 230 million and growing faster than previously predicted (2). The multicultural population of Korea has also increased, reaching 367,775 households as per the 2020 census. This accounts for 2.1% of the total population, which is an increase of 3.9% compared to 2019 (3). The number of children from multicultural families has also increased significantly from 44,258 in 2007 to 264,626 in 2019 (4).

Immigrants lack resources and experience poor socioeconomic conditions in general, which may negatively affect their health (5). Immigrants have limited relational networks, as most of their social capital remains in their place of origin (5). It is reported that differences in access to health resources, obtaining health-related information, and helping neighbors in responding to emergencies are related to these health inequalities. Additionally, the recent health care crisis caused by COVID-19 is further accelerating the health inequality faced by immigrants with different cultural backgrounds (6). In particular, a lack of social capital may negatively impact the health of multicultural adolescents, as adolescents are largely dependent on their parents.

Social capital has been defined in various ways by researchers such as Bourdieu (7), Coleman (8), and Putnam (9). Consolidating previous definitions, Lin et al. (10) defined social capital as resources embedded in the social structure, accessible to individuals, and usable for purposive actions. In the field of health promotion, social capital is regarded as a protective factor for health. Social capital correlates with socioeconomic inequalities in health; it buffers negative health effects and positively impacts the health of people with low socioeconomic status (SES) (11). At the individual level, it can enhance emotional and financial support, either by offsetting the negative effects of stress or by improving stress coping ability (12). Health behavior has also been shown to mediate social capital and health (13). At the community level, social capital can influence an individual's health through social networks and norms (11).

As health inequality established in childhood and adolescence can continue into adulthood (14), it is necessary to pay attention to adolescents' health determinants. Especially during adolescence, individuals' educational levels and social networks increase, and neighborhood and social factors, such as peer influence, play a prominent role in health (15). Therefore, it is meaningful to explore the relationship between adolescent social capital and adolescent health, rather than simply examining the correlation between SES and adolescent health. Strengthening multicultural adolescents' social capital can bridge health inequalities in their adulthood.

According to the World Health Organization (16), depression is the leading cause of illness and disability among adolescents worldwide. Unipolar depressive disorder is the leading cause of disability-adjusted life years (DALYs) lost in adolescents, and suicide is the third leading cause of death during adolescence. In addition, depression in adolescence has been reported to be highly correlated with emotional disorders in adulthood (16). Most mental disorders common among adolescents remain undiagnosed and untreated and pose the risk of serious consequences in adulthood. In particular, multicultural adolescents report more depressive symptoms than the general population of adolescents (17, 18). In a previous longitudinal analysis of multicultural adolescents in Korea, it was reported that the trajectory of depressive symptoms gradually increased (19).

As confirmed in previous systematic reviews, there is sufficient evidence for the relationship between social capital and mental health (11, 20). Specifically, a longitudinal study using data from the Korea Youth Panel Survey (KYPS) showed that there were fewer adolescents with depressive symptoms from families with high levels of social capital (21).

Based on empirical evidence that social capital is related to adolescent depressive symptoms, we aimed to determine whether there are certain patterns or combinations in the social capital of multicultural adolescents and whether such combinations create differences in health outcomes. To this end, we used latent profile analysis (LPA), which is an exploratory person-centered approach to investigate subgroups and patterns within populations, unlike a variable-centered approach (22). This approach is useful for identifying the nature of social capital among multicultural adolescents and for explaining how it translates into health outcomes. Although there is no clear standard for measuring adolescents' social capital, it is measured based on their relationships within the family, peer groups, school, and community (23–25). Previous studies using data from the Korean Children's Panel Survey used family social capital, school social capital (friends and teachers), and community social capital (24, 25) as adolescents' social capital indices. However, previous studies that measured adolescents' social capital had limitations in that they did not include policy support and out-of-school networks, which are important components of community social capital for multicultural adolescents. For example, Healthy Family and Multicultural Family Support Centers in Korea operate parent education and counseling, home visit services, case management, family activities, and cultural programs (26), which is a large axis of community social capital for multicultural adolescents. Therefore, this study used family social capital, school social capital, and community social capital as multicultural adolescents' social capital indices based on previous studies (24, 25), and considering the limitations of previous studies, policy support and out-of-school networks were additionally included as community social capital indices.

The purpose of the present study was to identify the profile of social capital using LPA among multicultural adolescents in Korea and examine the sociodemographic characteristics associated with these profiles. In addition, this study aimed to examine the association between these social capital profiles and depressive symptoms.

## Method

### Data Set and Sample

We used data from the Multicultural Adolescents Panel Survey (MAPS) conducted by the National Youth Policy Institute (NYPI) in Korea. When the first panel was established in 2011, the research population of MAPS comprised multicultural students in the 4^th^ grade of elementary school in Korea. A total of 1,635 adolescents (35%) were sampled in the first year from the 4,452 multicultural students attending 2,537 elementary schools nationwide. MAPS consists of panel data tracked annually since 2011, and the original sample retention rate was 81.2% in 2016. Additional information on MAPS can be found at https://www.nypi.re.kr/archive.

This study was aimed at adolescents in the 3^rd^ year of middle school (roughly equivalent to early secondary or high school in most international educational systems). Since the gap between regions and schools widens upon entering high school, the third year of middle school is suitable for exploring the possibility of resolving the gap through social capital (27). Therefore, this study used MAPS data from the 6^th^ year, conducted in 2016. Out of a total of 1,329 adolescents surveyed in 2016, 1,244 were included in the analysis after excluding 18 who did not respond to the questionnaire, 63 incomplete responses, and 4 respondents whose both parents were immigrants. Data excluding personal identifiers were obtained upon submitting a research plan to the National Youth Policy Institute. This study was conducted with the approval of the researcher's institution (IRB no. 2020R0011-001).

### Measures

#### Social Capital

Social capital was measured as family social capital (single index), school social capital (four indices), and community social capital (three indices). Family social capital is defined as the family background and the quality of parent–child relationship (25); in this study, it was measured through parenting attitude. School social capital refers to the individual's relationships with the school, peers, and teachers, and is measured through mutual trust and intimacy (28); in this study, it was measured through peer relationships and relationships with homeroom teachers, for example, by asking respondents to state the number of close friends or helpers they have at school. Community social capital comprises the social and physical environment surrounding the adolescent, and it is a resource obtained through the stability of structures and the closeness of relationships among institutions and organizations within the local community (29); in this study, it was measured through awareness of the community, policy support, and helpers outside of school.

Parenting attitude was assessed using the parental behavior inventory developed by Huh (30) and modified by Lee et al. (31). The inventory has questions related to abuse and neglect. The MAPS has seven items on neglect and each item is rated on a four-point Likert scale ranging from 1 (not at all) to 4 (extremely). The items about neglectful attitudes are reverse-coded and a higher total score indicates a more positive parenting attitude. Kang reported a Cronbach's alpha value of 0.84, and Cronbach's alpha in the present study was 0.82 (32).

Peer relationships and relationships with homeroom teachers were assessed using the school adjustment scale developed by Min (33) and modified by Kim et al. (34). The scale comprises five items rated on a four-point Likert scale ranging from 1 (not at all) to 4 (extremely). A higher total score indicates a more positive relationship. Jeong (35) reported a Cronbach's alpha of 0.83 for both peer relationships and relationships with homeroom teachers, while the Cronbach's alpha values in this study were 0.64 and 0.89, respectively. To report the number of close friends, the participants responded to the question, “How many close friends do you have?” Additionally, the number of helpers at school other than the homeroom teacher was assessed through the question, “Is there is an adult who can help when a difficult situation arises at school?” Responses were classified into seven categories: teachers in other classes, school nurses, counselors, multicultural teachers, subject teachers, guardians, and others. Each type was coded as 1 or 0 depending on the presence or absence of a specific type of helper, and the total score ranged from 0 to 7.

Awareness of the community was assessed using five questions from the National Longitudinal Study of Adolescent to Adult Health, conducted in the United States, also known as Add Health (36). The selected questions concerned neighborhood contentedness and were translated and modified by Kim et al. (34). The scale consisted of six items rated on a four-point Likert scale ranging from 1 (not at all) to 4 (extremely). The total score ranged from 4 to 24, with a higher total score indicating a higher awareness of the community. Kang (37) reported a Cronbach's alpha value of 0.73, while it was 0.75 in this study.

Service for supporting multicultural families was assessed through participants' experience of receiving 14 kinds of multicultural family policy support. Each type was coded as 1 or 0 and the total score ranged from 0 to 14. Helpers outside of school, which referred to helpers other than family members and school teachers, were identified through the question, “Is there is an adult who can help when you face a difficult situation outside of school?” The responses were classified into seven types: teachers in cram school, study room teachers, teachers at various youth facilities, neighborhood adults, mentors, tutors, and others. Each type was coded as 1 or 0 and the total score ranged from 0 to 7.

#### Depressive Symptoms

Depressive symptoms were assessed using a modified version of the Symptom Checklist-90-Revised (SCL-90-R) developed by Derogatis et al. (38) and standardized by Kim et al. (39). Three items were excluded from the 13-item SCL-90-R, and the 10 items on depressive symptoms used in the Korean Children and Youth Panel Survey (CYPS) were used. Each item was rated on a four-point Likert scale ranging from 1 (not at all) to 4 (extremely), with a higher total score indicating a higher level of depressive symptoms. Han and Kahng (40) reported a Cronbach's alpha value of 0.90, while it was 0.91 in this study.

#### Demographic Characteristics

The following demographic characteristics were used in the present study: gender, age, geographic location (metropolis, urban, rural), monthly household income, education level of parents, and country of origin of immigrant parent (Korea, China, South-east Asia, Japan, and others).

### Statistical Analysis

An LPA was performed to identify the latent profile types of social capital. To determine the optimal class solution, various class models, such as Bayesian information criterion (BIC) and sample size adjusted BIC (saBIC), were compared in information criterion (IC)-based fit indices. Lower values of this index indicate better model fit. Entropy was also checked and values above 0.80 indicated adequate classification accuracy (41). We also compared improvements between k-class and k-1 class models using the Lo-Mendell-Rubin likelihood-ratio test (LMR) and the bootstrap likelihood-ratio test (BLRT). Significant *p*-values suggest that the inclusion of additional classes improved the fit (42). Chi-square test and analysis of variance (ANOVA) were used to investigate whether sociodemographic and clinical characteristics discriminate between classes. To compare the level of depressive symptoms across identified classes, Quade's non-parametric ANCOVA was performed to control for sociodemographic variables. Statistical analysis was performed using SPSS 23.0 (IBM, New York, USA) and Mplus 8.0 (Muthen & Muthen, Los Angeles, USA).

## Results

The exploration of the types of social capital for multicultural adolescents showed that the model converged with a 4-profile model (Table 1). The number of groups was determined based on the model fit indices and total correct classification rate (43, 44). BIC and saBIC decreased with increasing number of classes and these indices were lowest in the 5-profile model. Entropy converged to 1 as the number of potential groups increased. LMR and BLRT were statistically significant in the 2-profile and 4-profile models, confirming the existence of different heterogeneous groups in social capital. LMR was not statistically significant in the 3-profile and 5-profile models. The 4-profile model had lower BIC and saBIC values than the 2-profile model and the LMR and BLRT showed that both models supported the alternative hypothesis (*p* < 0.001). Focusing on the 2-profile and 4-profile models, it was confirmed that the information and the distribution rates were at least 5%. The 4-profile model had more information than the 2-profile model, and the classification rate was 6.1–67.7%, indicating high practical usefulness. Therefore, a 4-profile model was the most suitable. The final Average Latent Class Probabilities of the 4-profile model range from 0.872 to 0.944, which is close to 1.0, indicating that the classification accuracy was high (45).

**Table 1:** Model fit indices of latent profile analysis and distribution rate of social capital (*N* = 1,244).

**Model**	**Model fit indices**					**Latent class distribution rate (%)**				
	**BIC**	**saBIC**	**LMR**	**BLRT**	**Entropy**	**1**	**2**	**3**	**4**	**5**
2-profile	40737.91	40658.50	<0.001	<0.001	0.76	74.0	26.0			
3-profile	40210.22	40102.22	0.582	<0.001	0.85	71.6	6.3	22.1		
4-profile	39892.01	39755.43	0.019	<0.001	0.87	19.5	6.7	6.1	67.7	
5-profile	39683.11	39517.94	0.178	<0.001	0.91	1.6	8.4	68.9	0.8	20.3

*BIC, Bayesian Information Criteria; saBIC, Sample-size Adjusted BIC; LMR, Lo-Mendell-Rubin adjusted Likelihood Ratio Test; BLRT, Parametric Bootstrapped Likelihood Ratio Test*.

### Social Capital Latent Profile

**Figure 1:**
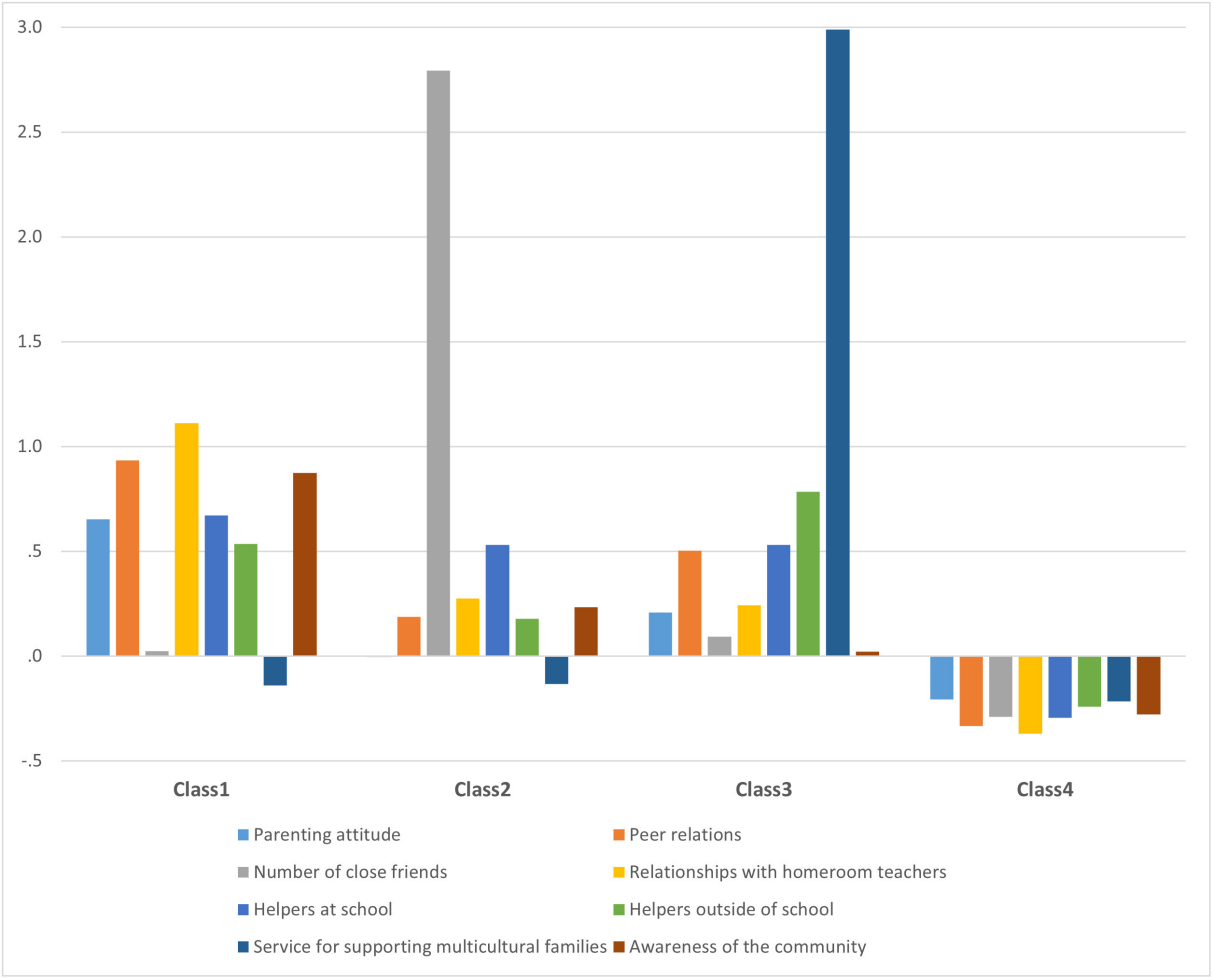
Illustration of z-score distribution of social capital in the four profiles defined in the latent profile analysis.

Results grouped by social capital profile are shown in Figure 1 and Table 2. Class 1 (*n* = 243, 19.5%) was characterized by the number of close friends; service for supporting multicultural families was close to the average (standard score 0) and other types of social capital were high. Class 2 (*n* = 83, 6.7%) was characterized by parenting attitudes; service for supporting multicultural families was close to the average (standard score of 0) and the number of close friends was high. Class 3 (*n* = 76, 6.1%) was characterized by the number of close friends; awareness of the community converged to the average (standard score of 0) and service for supporting multicultural families was high. Class 4 (*n* = 842, 67.7%) showed low levels of all types of social capital.

**Table 2:** Differences in indices of social capital among latent classes (*N* = 1,244).

**Group indices**	**Total** **(*n* = 1,244)** ***M* ± *SD***	**Class 1** **(*n* = 243)** ***M* ±*SD***	**Class 2** **(*n* = 83)** ***M* ± *SD***	**Class 3** **(*n* = 76)** ***M* ±*SD***	**Class 4** **(*n* = 842)** ***M* ± *SD***	***F* (*p*)**	***Post hoc* (Scheffé)**
Parenting attitude	22.82 ± 3.38	25.03 ± 2.99	22.81 ± 3.75	23.53 ± 3.59	22.13 ± 3.14	53.45 (<0.001)	2, 3, 4 <1
Peer relations	14.38 ± 1.79	16.05 ± 1.55	14.71 ± 1.45	15.28 ± 2.00	13.78 ± 1.50	147.42 (<0.001)	4 <2, 3 <1
Number of close friends	7.51 ± 5.71	7.64 ± 3.44	23.46 ± 6.60	8.04 ± 3.90	5.86 ± 3.36	568.30 (<0.001)	4 <1, 3 <2
Relationships with homeroom teachers	15.52 ± 2.82	18.66 ± 1.73	16.30 ± 2.88	16.21 ± 2.51	14.48 ± 2.34	215.96 (<0.001)	4 <2, 3 <1
Helpers at school	0.95 ± 1.15	1.73 ± 1.41	1.57 ± 1.48	1.57 ± 1.25	0.62 ± 0.83	92.22 (<0.001)	4 <1, 2, 3
Helpers outside of school	0.46 ± 0.65	0.81 ± 0.82	0.58 ± 0.63	0.97 ± 0.88	0.30 ± 0.50	64.65 (<0.001)	4 <2 <1, 3
Service for supporting multicultural families	0.40 ± 0.91	0.27 ± 0.51	0.28 ± 0.57	3.11 ± 1.41	0.20 ± 0.47	577.45 (<0.001)	1, 2, 4 <3
Awareness of the community	17.59 ± 2.99	20.21 ± 2.80	18.29 ± 3.28	17.66 ± 3.34	16.76 ± 2.49	106.59 (<0.001)	4 <2, 3 <1

### Participants' Sociodemographic Characteristics

The demographic characteristics of multicultural adolescents included in this study are shown in Table 3. Among the total respondents, 49.0% (*n* = 610) were boys and 51% (*n* = 634) were girls; the mean age was 14.97 years (*SD* = 0.36). A total of 555 multicultural adolescents (44.6%) lived in urban areas and belonged to households with a mean monthly income of approximately KRW 2.55 million. A total of 1,203 (96.7%) multicultural adolescents had an immigrant mother and 41 (3.3%) had an immigrant father. The most common country of origin of mothers was Japan, followed by South-east Asia, and China. The most common country of origin for fathers was also Japan. Most fathers were high school graduates, followed by below middle school graduates, university graduates and above, and college graduates. In the case of mothers, most were high school graduates, followed by college graduates, university graduates and above, and middle school graduates and below.

**Table 3:** Differences in the characteristics of participants according to the profile group (*N* = 1,244).

**Characteristics**	**Categories**	**Total** ***n* (%) or** ***M* ±*SD***	**Class 1** ***n* (%) or** ***M* ± *SD***	**Class 2** ***n* (%) or** ***M* ±SD**	**Class 3** ***n* (%) or** ***M* ± *SD***	**Class 4** ***n* (%) or** ***M* ±*SD***	**χ^2^ or *F* (*p*)**
Gender	Boy Girl	610 (49.0) 634 (51.0)	126 (51.9) 117 (48.1)	55 (66.3) 28 (33.7)	31 (40.8) 45 (59.2)	398 (47.3) 444 (52.7)	13.75 (0.003)
Age (years)		14.97 ± 0.36	14.95 ± 0.42	14.94 ± 0.29	14.92 ± 0.36	14.98 ± 0.34	1.21 (0.305)
Geographic location	Metropolis Urban Rural	320 (25.7) 555 (44.6) 369 (29.7)	55 (22.6) 98 (40.3) 90 (37.0)	18 (21.7) 42 (50.6) 23 (27.7)	26 (34.2) 33 (43.4) 17 (22.4)	221 (26.2) 382 (45.4) 239 (28.4)	11.83 (0.066)
Monthly household income (10,000 KRW)		255.83 ± 113.97	263.71 ± 128.74	244.94 ± 103.90	230.86 ± 92.76	256.89 ± 111.90	1.89 (0.130)
Educational level of father	Middle School and below High school College University and above	381 (30.6) 643 (51.7) 85 (8.6) 135 (10.9)	75 (30.9) 126 (51.9) 12 (4.9) 30 (12.3)	31 (37.3) 39 (47.0) 6 (7.2) 7 (8.4)	22 (28.9) 41 (53.9) 5 (6.6) 8 (10.5)	253 (30.0) 437 (51.9) 62 (7.4) 90 (10.7)	4.45 (0.879)
Educational level of mother	Middle School and below High school College University and above	141 (11.3) 583 (46.9) 318 (25.6) 202 (16.3)	27 (11.1) 112 (46.1) 56 (23.0) 48 (19.8)	10 (12.0) 43 (51.8) 21 (25.3) 9 (10.8)	6 (7.9) 35 (46.1) 20 (26.3) 15 (19.7)	98 (11.6) 393 (46.7) 221 (26.2) 130 (15.4)	6.41 (0.669)
Type of multicultural family	Father Mother	41 (3.3) 1,203 (96.7)	7 (2.9) 236 (97.1)	3 (3.6) 80 (96.4)	0 (0.0) 76 (100.0)	31 (3.7) 811 (96.3)	3.14 (0.370)
Father's country of origin	Korea China South-East Asia Japan Others	1,203 (96.7) 2 (0.2) 6 (0.5) 17 (1.4) 16 (1.3)	236 (97.1) 1 (0.4) 2 (0.8) 1 (0.4) 3 (1.2)	80 (96.4) 0 (0.0) 0 (0.0) 2 (2.4) 1 (1.2)	76 (100.0) 0 (0.0) 0 (0.0) 0 (0.0) 0 (0.0)	811 (96.3) 1 (0.1) 4 (0.5) 14 (1.7) 12 (1.4)	7.39 (0.766)
Mother's country of origin	Korea China South-East Asia Japan Others	41 (3.3) 311 (25.0) 386 (31.0) 445 (35.8) 61 (4.9)	7 (2.9) 62 (25.5) 70 (28.8) 84 (34.6) 20 (8.2)	3 (3.6) 17 (20.5) 29 (34.9) 32 (38.6) 2 (2.4)	0 (0.0) 14 (18.4) 23 (30.3) 36 (47.4) 3 (3.9)	31 (3.7) 218 (25.9) 264 (31.4) 293 (34.8) 36 (4.3)	16.88 (0.154)

### Sociodemographic Characteristics Across Identified Classes

Examining the sociodemographic characteristics of multicultural youth among the four identified social capital types, we found a significant difference with regard to gender (χ^2^ = 13.75, *p* = 0.003). However, there was no significant difference with respect to age, geographic location, monthly household income, type of multicultural family, country of origin of immigrant parent, and education level of parents as shown in Table 3.

### Depressive Symptoms According to Identified Latent Classes

The mean score for depressive symptoms among multicultural adolescents was 17.07 ± 5.34. Examination of the differences in the depressive symptoms between the groups based on the social capital profile, while controlling for demographic variables, showed statistically significant differences between the groups (*F* = 44.33, *p* < 0.001). The pairwise comparisons showed that Class 1 had a significantly lower score on depressive symptoms than Classes 2, 3, and 4. Class 4 had the highest depressive symptoms score. There was no statistically significant difference between the depressive symptoms scores of Classes 2 and 3 (see Table 4).

**Table 4:** Comparison of depressive symptoms between the four classes (*N* = 1,244).

**Variables**	**Total** **(*n* = 1,244)** ***M* ± *SD***	**Class 1** **(*n* = 243)** ***M* ± *SD***	**Class 2** **(*n* = 83)** ***M* ± *SD***	**Class 3** **(*n* = 76)** ***M* ± *SD***	**Class 4** **(*n* = 842)** ***M* ± *SD***	***F* (*p*)**	***Post hoc* (Bonferroni)**
Depressive symptoms	17.07 ± 5.34	13.91 ± 4.43	16.49 ± 5.27	16.74 ± 4.95	18.07 ± 5.25	44.42 (<0.001)	1 <2 = 3 <4

*Covariates (gender, age, education level of parents, foreign parent's country of origin, income) were included in non-parametric ANCOVA*.

## Discussion

The present study examined the profile of social capital and its association with depressive symptoms and self-rated health among multicultural adolescents in Korea. We identified the latent profiles of multicultural adolescents' social capital using the 6^th^ MAPS data.

Our analysis revealed homogeneous groups that exist in terms of social capital in Korea's multicultural adolescent population. Four profiles were identified and their distribution reflected that many multicultural adolescents lacked social capital. Overall, Class 1 had high social capital and Class 4 had poor social capital. More than two-thirds of multicultural adolescents belonged to Class 4 while only approximately one-fifth belonged to Class 1. Classes 2 and 3 comprised 6.7 and 6.1% of the total multicultural adolescents, respectively. It is difficult to make a direct comparison due to different indices, but these results were slightly different from the findings of Yoo's (25) study, which also classified the social capital profiles of multicultural adolescents. In Yoo's (25) study, three latent profiles were identified: high overall social capital (24.9%), average social capital (65.7%), and school social capital vulnerability (9.4%); more than half of multicultural adolescents had moderate social capital. However, in the present study, most multicultural adolescents fell into the low social capital group. This can be explained by the social capital indices that were not included in previous studies, that is, helpers at school other than the homeroom teacher, helpers outside of school, and policies supporting multicultural families. Previous studies' conceptualization of adolescents' social capital was limited to social capital in the family, relationships with friends and teachers, and community awareness (21, 23, 25). This study found that Class 3 had a very high level of out-of-school helpers and service for supporting multicultural families. Furthermore, it was found that the beneficiary level of policy services differed greatly among multicultural adolescents in this study. With the exception of Class 3, which accounted for only approximately 6%, the level of multicultural-related policy support was very low in other classes, indicating that the overall scope of policy support was narrow. It also means that the benefits were concentrated on some multicultural adolescents. The level of out-of-school helpers in both Classes 1 and 3 was high, but perhaps the out-of-school helpers in Class 3 were more likely to include supporters of multicultural support centers, while those in Class 1 were more likely to be community neighbors. In this study, the detailed classification could not be confirmed because only the numerical value was measured. Therefore, it is necessary to distinguish the specific type of out-of-school helpers in further research.

Parenting attitude was highest in Class 1, followed by Classes 3, 2, and 4. The number of close friends was very high in Class 2, but in terms of peer relationship, Class 1 was at the top, followed by Classes 3, 2, and 4. The overall high social capital of Class 1 can be explained by the interaction of social capital at home and school. In other words, parental participation and adolescents' sociality are related to each other which is an important prerequisite for building adolescents' social capital (46). In a previous study with Swedish adolescents, those with low trust and sense of belonging to family and school tended to find strong social networks among their peers outside of school (23). However, as we only measured out-of-school helpers and not out-of-school peers, it was difficult to confirm these characteristics. The relationships with the homeroom teacher and helpers at school other than the homeroom teacher were highest in Class 1, followed by similar figures in Classes 2 and 3, and the lowest in Class 4. The score representing the relationship with the homeroom teacher in Classes 2 and 3 was lower than that in Class 1, but networking with other helpers (e.g., teachers of specific subjects, school nurses, etc.) at the school was not poor.

After controlling for sociodemographic variables, the level of depressive symptoms differed across the identified profiles. These results are in line with previous studies showing that social capital is associated with depressive symptoms (47). The present study showed that the level of depressive symptoms was different depending on the profile of social capital. Multicultural adolescents with high social capital had low levels of depressive symptoms, while those with low social capital had a high level of depressive symptoms. In particular, Class 4 was the lowest in all eight indices constituting social capital in this study and showed the highest depressive symptoms. Exploring the reason for the high levels of depressive symptoms among multicultural adolescents from Class 4 was not the focus of this study. Nevertheless, social capital can be a means of creating an environment that supports the mental health of multicultural adolescents through the social networks surrounding them (48); however, the result of this study suggests that this buffer might not exist for Class 4 adolescents or that it may be difficult for them to access for them. Therefore, there is a need for careful assessment and intervention in the depressive symptoms of multicultural adolescents with low levels of social capital.

This study demonstrates that all types of social capital available to multicultural adolescents, such as family, friends, teachers, helpers inside and outside school, and multicultural policy support, can be the starting point for early intervention to address the mental health issues faced by multicultural adolescents. Previous studies found that trust at the individual level was negatively related with depressive symptoms (47). Trust in neighbors was shown to be negatively related to depression, but individual participation in community activities was not associated with depression. In this study, instead of including trust or participation in community activities at the individual level, social capital was identified by focusing on various networks surrounding multicultural adolescents. Networking social capital may positively impact health through expressive and instrumental resources accessible through social connections (48). Multicultural adolescents, who act as the cultural and language translators for their foreign parents, may confront conflicts between their role as children at home and family mediators between their foreign parents and the dominant culture (49, 50). Social capital accumulated from social relations can serve as a buffer to reduce stress for multicultural adolescents in this situation (51). However, there was no statistically significant difference in the level of depressive symptoms between Classes 2 and 3 despite the differences in social capital indices. Although the cause could not be confirmed in this study, it is necessary to explore the reasons in future studies.

Regarding sociodemographic factors associated with the identified classes, no other factor except gender differed between the groups defined by social capital profiles. While Classes 1 and 4 had an equal proportion of boys and girls, there were more boys in Class 2, and relatively more girls in Class 3. Dufur et al. (52) reported that boys and girls did not seem to build social capital in different ways. Nevertheless, girls received more educational returns from social capital than boys in Dufur et al.'s study. In the present study, there was a slight difference in the gender ratio between Classes 2 and 3, but there was no significant difference in the level of depressive symptoms. However, the gender differences between Dufur et al.'s study and the present study cannot be interpreted in the same manner, as they may differ in the way social capital influences academic achievement and health outcomes and because of the difference in the methodological approach used in the two studies, with the former being variable-centered and the latter being person-centered.

The current findings have some implications for intervention, policy, and future research. First, it is necessary to pay close attention to the formation of social capital of multicultural adolescents, as they are relatively disadvantaged in accumulating social capital. The fact that approximately two-thirds of all multicultural adolescents in Korea lack social capital in general indicates that they may experience alienation. In particular, due to the recent COVID-19 pandemic, relationships with peers and teachers have been cut off (e.g., social distancing and lockdowns) (53), and support for multicultural families provided in the community is also limited (26), which is expected to further lower the level of social capital of multicultural adolescents. Future research should examine the relationship between social capital and depressive symptoms in multicultural adolescents during the pandemic once the MAPS data collected during the pandemic becomes available. Second, when assessing the depressive symptoms of multicultural adolescents, it is necessary to investigate all aspects of social capital (i.e., family, school, and community social capital) to identify the leverage points. In particular, in the case of community social capital of multicultural adolescents, since community centers play an important role for multicultural support, it is necessary to examine policy support and out-of-school networks. Social capital has a multi-level effect on depression among multicultural adolescents (11, 20). When planning a depression intervention program for multicultural adolescents, it may be useful to consider ways to utilize the social capital they have or find ways to make up for the lack of social capital.

The present study has some limitations. First, the attributes of social capital were not strictly classified and measured because only the variables measured in the panel study were used in this study. Only the network aspects of out-of-school helpers and service for supporting multicultural families were considered, and trust could not be measured. In future studies, if bridging social capital and bonding social capital are separately measured, the differences and effects of each type of social capital can be identified more clearly. Second, only the number of networks of multicultural adolescents was identified, but the structure of the networks was not explored. Further research aimed at assessing the extent of the network is needed. Third, our study used secondary data collected through self-reported measures. Therefore, limitations such as some factors related to adolescents' social capital not being included (e.g., parent-level social capital) and objective indicators (e.g., diagnosed depression) being insufficient must be considered. Fourth, because of the heteroscedasticity of the data, a non-parametric test was performed to confirm the difference in outcome by class. Finally, since the present study was cross-sectional, cause-effect relationship could not be confirmed. Despite the complex reciprocal effects between depression and social capital (54, 55), our study took a sociological approach to the mental health of multicultural adolescents.

Despite these limitations, the present study helps in understanding the phenomenon by defining the social capital of multicultural adolescents more broadly. In addition, this study has significance as basic data for policies to promote the social capital of multicultural adolescents and prevent depressive symptoms among this population.

## Conclusions

This study attempted to classify the latent classes of multicultural adolescents according to social capital using the Multicultural Adolescents Panel Study (MAPS) data and identify the differences in depressive symptoms according to the social capital profile of adolescents. Eight indices including parenting attitude, peer relationship, relationship with homeroom teachers, number of close friends, helpers at school, awareness of the community, and service supporting multicultural families were used to assess the social capital of multicultural youth. As a result, four latent classes were identified based on the social capital of multicultural adolescents. There was a difference in the level of depressive symptoms according to the social capital profile. Therefore, differences in social capital must be considered when providing support to reduce depression among multicultural adolescents.

## Data Availability Statement

The raw data supporting the conclusions of this article will be made available by the authors, without undue reservation.

## Ethics Statement

The studies involving human participants were reviewed and approved by University of Ulsan Institutional Review Board. This was a secondary data study conducted by receiving data, excluding personal identifiers from the National Youth Policy Institute. Written informed consent from the participants' legal guardian/next of kin was not required to participate in this study in accordance with the national legislation and the institutional requirements.

## Author Contributions

EK and MS: conceptualization, methodology, data curation, and writing—original draft/review and editing. MS: formal analysis, visualization, resources, and funding acquisition. Both authors contributed to the article and approved the submitted version.

## Funding

This work was supported by the 2021 Research Fund of the University of Ulsan.

## Conflict of Interest

The authors declare that the research was conducted in the absence of any commercial or financial relationships that could be construed as a potential conflict of interest.

## Publisher's Note

All claims expressed in this article are solely those of the authors and do not necessarily represent those of their affiliated organizations, or those of the publisher, the editors and the reviewers. Any product that may be evaluated in this article, or claim that may be made by its manufacturer, is not guaranteed or endorsed by the publisher.
